# An overview of food safety and bacterial foodborne zoonoses in food production animals in the Caribbean region

**DOI:** 10.1007/s11250-016-1082-x

**Published:** 2016-05-23

**Authors:** Maria Manuela Mendes Guerra, Andre M. de Almeida, Arve Lee Willingham

**Affiliations:** Ross University School of Veterinary Medicine, P.O. Box 334, Basseterre, St. Kitts and Nevis

**Keywords:** Caribbean region, Food animal products, Foodborne diseases, Livestock, Bacterial zoonosis, Surveillance

## Abstract

Foodborne diseases (FBDs) in the Caribbean have a high economic burden. Public health and tourism concerns rise along with the increasing number of cases and outbreaks registered over the last 20 years. *Salmonella* spp., *Shigella* spp., and *Campylobacter* spp. are the main bacteria associated with these incidents. In spite of undertaking limited surveillance on FBD in the region, records related to bacterial foodborne zoonoses in food-producing animals and their associated epidemiologic significance are poorly documented, giving rise to concerns about the importance of the livestock, food animal product sectors, and consumption patterns. In this review, we report the available published literature over the last 20 years on selected bacterial foodborne zoonoses in the Caribbean region and also address other food safety-related aspects (e.g., FBD food attribution, importance, surveillance), mainly aiming at recognizing data gaps and identifying possible research approaches in the animal health sector.

## Introduction

Foodborne diseases (FBDs) are a serious global health problem. Microbial food safety is an increasing public health concern worldwide. Each year, as many as 600 million, or almost one in 10 people in the world, fall ill after consuming some sort of contaminated food. Of these, 420,000 people die, including 125,000 children under 5 years of age as stated in the World Health Organization’s estimates on the global burden of foodborne diseases (WHO 2015). According to the CDC (2015), in the USA in 2013, 818 foodborne disease outbreaks were reported, resulting in 13,360 illnesses, 1062 hospitalizations, 16 deaths, and 14 food recalls. In the European Union (EU), in 2012, a total of 5363 foodborne outbreaks were reported, resulting in 55,453 human cases, 5118 hospitalizations, and 41 deaths (EFSA 2014). The most frequent causes of foodborne illness were diarrheal disease agents, particularly norovirus and *Campylobacter* spp. Foodborne diarrheal disease agents caused 230,000 (95 % UI 160,000–320,000) deaths, particularly non-typhoidal *Salmonella enterica* (NTS) which causes diarrheal and invasive disease (WHO 2015).

Even though these pathogens usually cause mild to moderate self-limiting gastroenteritis, invasive diseases and complications may occur, resulting in more severe cases. In the USA, the pathogen-food category pairs responsible for most outbreak-associated illnesses are *Salmonella* in chicken, *Salmonella* in pork, and *Salmonella* in seeded vegetables (CDC 2013). Evidence of FBD in low- and middle-income countries is still limited, but recent studies suggest that the most significant also comes from biological hazards (Grace 2015), with an estimated 20 % of all human illness and death associated with endemic zoonoses (Grace et al. 2011). This is particularly true in Africa and the Middle East where few countries have surveillance programs. By contrast, all Central/South American and Caribbean countries have some forms of notification system. With respect to the Caribbean, the regional surveillance data in this region from 2005 to 2014 points to FBD as an increasing public health concern as reflected by the growing number of cases and outbreaks reported to the official agencies. *Salmonella*, *Shigella*, and *Campylobacter* prevail among bacterial causative agents and contribute to the overall annual economic costs of syndromic acute gastroenteritis (AGE) and FBD, with an estimated burden of $US2.2 M and 40.4 M, respectively (Indar et al. 2015a, b). Considering animal health, the burden of animal and zoonotic infectious diseases in the Caribbean region is considered to be high. However, it is recognized that the majority of Caribbean countries lack the capacity to accurately recognize, diagnose, and respond to such diseases (ACP 2013). In spite of the surveillance system on FBD in the Caribbean region, records related to bacterial foodborne zoonoses in food-producing animals and their associated epidemiologic significance are poorly documented. Aiming to assess the knowledge gaps in relation to this particular topic, and also to contribute to the identification of relevant areas where meaningful research may be conducted in the future, this overview covers (1) the evolution of food-producing animals in the region during the last two decades, (2) the main bacteria associated with FBD in the Caribbean, (3) food attribution with FBD in the Caribbean, (4) the importance of FBD to the Caribbean region and bacteria prevalence studies in food-producing animals and food products of animal origin in the Caribbean, (5) sources of animal and food contamination, and (6) the need for integrated animal-human surveillance of zoonotic foodborne pathogens.

## Material and methods

A literature search was conducted on the available documentation published in English, Portuguese, and Spanish regarding bacterial foodborne zoonoses and food safety in the Caribbean Region from 1995 to 2015. Relevant studies were identified using a combination of key words in multiple databases, including PubMed, Science Direct, ISI Web of Science, and Google Scholar. The reference lists of relevant papers were searched for additional studies not found through database searching. We looked for studies on the most important foodborne bacterial hazards of current concern as considered by EDES c/o COLECAP (2012), *Salmonella* spp., *Campylobacter* spp., and verotoxigenic *Escherichia coli*. The combination was made of general terms related to bacterial studies and risk factors associated with food-producing animals (i.e., animal husbandry, farm animals, livestock, burden, meat, milk, eggs, public health, food safety, tourism) and each of the bacteria terms. Each search term was always used in combination with “Caribbean.”

The countries of the Caribbean subregion, as defined for the purposes of this study, comprise the full member states of the Caribbean Community organization (Caricom), Antigua and Barbuda, the Bahamas, Barbados, Belize, Dominica, Grenada, Guyana, Haiti, Jamaica, Monserrat, Saint Kitts and Nevis, Saint Lucia, Saint Vincent and the Grenadines, Trinidad and Tobago, and Suriname. Except for Belize, Guyana, and Suriname, these are all island states (Caricom Member States, Caricom 2015).

## Results and discussion

### Food-producing animals and meat production in the Caribbean region

In the Caribbean, livestock production varies in importance across the region depending on environment and culture. Production and consumption figures reflect subregion-specific livestock species and products. For example, Jamaica has the biggest livestock sector in the region, with well-developed poultry and other livestock industries (FAO 2007). Small ruminants, sheep and goats, are considered very important species in the Caribbean, not only for domestic meat production but also as a source of emergency income (Vokaty and Torres 1997). Among the livestock products produced in the region, meat has the highest production figures. However, the production of the Caribbean accounts for only 0.1 % of total world meat production with these countries importing far more live animals and primary livestock products than they export (FAO 2007). This region is a net importer of meat, as local production is insufficient to meet demand, particularly that created by the tourist industry (Vokaty and Torres 1997). The overall populations of live animals of the major livestock species in the Caribbean in the years 1993 and 2013 are provided in Table 1 to show the evolution of livestock keeping during the past two decades. In the region, meat from poultry leads the industry, followed by pork and beef (FAOSTAT 2015). The amounts of food items produced from the major livestock species in the Caribbean for those same years are presented in Table 2 (FAO 2007).

**Table 1:** Number of heads (average) of live animals of major livestock species in the Caribbean—comparison years 1993/2013

Livestock live animals	1993	2013
Cattle	9325 K	9315 K
Goats	2234 M	3508 M
Pigs	3877 K	3542 K
Poultry	1118 K	2808 K
Sheep	3777 M	2587 M

*Source*: FAOSTAT 2015

*M* million, *K* thousand

**Table 2:** Production of food items from the major livestock species in the Caribbean—comparison years 1993/2013

Livestock primary items	1993	2013
Eggs—primary	152,623 K	253,085 K
Milk—cow	14,588 K	17,583 K
Milk—total	16,182 K	19,753 K
Meat—beef	224,889 K	238,226 K
Meat—chicken	336,066 K	569,219 K
Meat—goat	7,385 K	11,802 K
Meat—pork	214,999 K	339,985 K
Meat—sheep	7,372 K	13,034 K
Meat—total	801,1 K	1,184,3 K

*Source*: FAOSTAT 2015

*K* thousand

The growth in livestock production in the last two decades reflects the regional trend of consumers increasingly moving away from the consumption of beef toward poultry, pork, eggs, and dairy products (FAO 2014), which is in line with global consumption trends. The exception is the consumption of mutton and goat that still prevails and continues to grow in the region, and which is mostly consumed by the local population rather than by visiting tourists. Generally, there is a preference for goat meat rather than mutton by Caribbean nationals (Vokaty and Torres 1997). Most likely, the consumption of meat and food animal products in the region will follow global projections for 2050 of a moderate increase in meat consumption, based mainly on increased consumption of pork and especially poultry, while the consumption of eggs and milk are expected to continue to increase and decrease, respectively (at least in developed countries) (Kearny 2010).

### Bacteria associated with FBD in the Caribbean

Pathogens associated with the majority of outbreaks in the USA and Europe also rank among the most common causes of outbreaks and FBD cases in the Caribbean, namely, those associated with AGE and diarrhea as seen in a set of studies aimed at estimating the burden of illness (BOI) in seven Caribbean countries (i.e., Trinidad and Tobago, Dominica, Guyana, St Lucia, Grenada, Barbados, Jamaica) from 2008 to 2013. *Salmonella* spp. and *Shigella* spp. were the most frequently found and with the highest estimated burdens after norovirus (Indar et al. 2015b). In Trinidad and Tobago in 2008 and 2009, Lakhan et al. (2013) related *Salmonella* and *Shigella*, along with rotavirus and norovirus, to AGE. In Dominica (Ahmed et al. 2013), *Salmonella* and *Shigella* were also related to AGE, with norovirus and *Giardia* as the leading etiologic agents. *Giardia* was also reported to be the prevalent etiologic agent in Guyana; however, no data was reported regarding bacterial agents due to a lack of laboratory capacity (Persuad et al. 2013). In St. Lucia and Grenada (Gabriel et al. 2013; Glasgow et al. 2013), *Salmonella* was the most commonly isolated enteropathogen associated to AGE, followed by rotavirus and norovirus, respectively. In Barbados, norovirus led the foodborne pathogens causing AGE-related illness, followed by *Salmonella* and *Campylobacter*. In Jamaica, the burden of illness study revealed that there was no determination of pathogen specific causes of AGE (Fletcher et al. 2013). Overall, underreporting of syndromic AGE in these countries ranged from 64 to 83 % and for laboratory—confirmed FBD pathogens from 74 to 99 % (Indar et al. 2015b).

In addition to these retrospective, cross-sectional studies, other reports on the frequency of enteropathogens in human fecal and rectal specimens indicate *Shigella*, *Salmonella*, and enteropathogenic *E. coli* as the most frequently found pathogens in Trinidad (Khan-Mohammed et al. 2005) and *Salmonella*, *Campylobacter*, and *Shigella* in Barbados (Kumar et al. 2014). Furthermore, the Caribbean Public Health Agency (CARPHA) has recently compiled data regarding FBD and *Salmonella* infections in 21 English-speaking Caribbean countries detected through regional surveillance in the years 2005–2014 (Indar et al. 2015a). During this period, the number of cases of reported human FBD in the Caribbean increased by 26 %. Non-typhoidal *Salmonella* was the most common pathogen causing FBD (47 %), followed by ciguatera poisoning (24 %), *Salmonella* typhi (9.8 %), *Shigella* (8 %), *Campylobacter* (6 %), and norovirus (3.9 %). There was an increase in non-typhoidal *Salmonella* (51 %), norovirus (26 %), and *Campylobacter* (25 %) from 2005 to 2014, while *Salmonella* typhi (−99 %), *Shigella* (−54 %), and ciguatera (−18 %) decreased during the same time period. *Salmonella* and *Shigella* were reported from more than half of the countries. *Campylobacter* and ciguatera were reported from three to six countries and norovirus from five to seven countries. A wider range of pathogens were reported from 2010 onward. *Salmonella* serotyping data indicated that while 146 serotypes were identified, enteritidis (29 %) and typhimurium (21 %) were the two most prevalent serotypes isolated from 2005 to 2014, followed by Mississippi (4.9 %). Enteritidis was the most commonly isolated in Trinidad and Tobago, Jamaica, and Suriname. Typhimurium was dominant in Barbados and Mississippi in Bermuda (Indar et al. 2015a).

### Food attribution with FBD in the Caribbean

Specific attribution studies in the Caribbean region are scarce. Indar-Harrinauth et al. (2001) published a case control study which provided evidence for the linkage of consumption of eggs with *Salmonella* enteritidis infection in Trinidad and Tobago. In the recently published WHO’s estimates of the global burden of foodborne diseases (2015), the expert elicitation results regarding sub-regional attribution estimates of the proportion of illnesses caused by *Campylobacter* spp., non-typhoidal *Salmonella* spp., and Shiga toxin-producing *E. coli* (STEC), through each exposure pathway in the Caribbean region, are undoubtedly related to food sources. *Campylobacter* illness is mainly associated with food sources (0.68 %) followed by contact with animals (domestic and wild; 0.11 %); non-typhoidal *Salmonella enterica* food source (0.49 %), animal contact (0.19 %), and human to human contact (0.15 %); and Shiga toxin-producing *E. coli* 0.53 % related to food sources, 0.17 % to direct contact with animals (domestic and wild), and 0.11 % related to human to human contact. Previously, in an attempt to overcome the deficiency of data, Pires et al. (2011) estimated the contribution of different food sources to human diseases using outbreak data available from Latin America and the Caribbean region between 1993 and 2010. Authors reviewed 6313 bacterial outbreaks reported by 20 countries and concluded that in general, the most important sources of bacterial disease were meat and dairy products, water, and vegetables in the 1990s, whereas eggs, vegetables, grains, and beans were the most important sources in the 2000s. The most frequently reported pathogens were *Staphylococcus aureus*, *Salmonella* spp., *E. coli*, and *Clostridium perfringens* in the 1990s, while *Salmonella* spp., *Vibrio parahemolyticus*, and *Shigella* spp. were the most frequently reported in the 2000s. The associated fluctuations observed for each pathogen between decades and countries were linked to changes in the control of zoonotic diseases, changes in food consumption habits, changes in public health focus, and changes in availability of data regarding different pathogens. The authors also identified many data gaps in the region and noted that some of the data does not represent the region totally but only some of the countries, considering the great variability in terms of reporting, namely, of the most frequently described pathogens. For example, FBD outbreak data from only two of the Caribbean countries, Bahamas and Barbados, were considered in the present overview; thus, the data can only be seen as an indicator of the reality in the Caribbean, based on geographical, cultural, and food consumption similarities.

### The importance of FBD in the Caribbean region

Apart from the substantial economic burden on the population and health care system imposed by FBD, the associated economic impact for nations that depend on tourism for financial support is also a reason of concern. Tourism is very important to the Caribbean economies, contributing about a third of the regional GDP. The region regularly and, in recent years, increasingly receives large numbers of passengers from cruise ships and also large numbers of visitors staying in hotels and resorts many of them with all inclusive meal plans. During the first 6 months of 2015, the Caribbean experienced a 7 % increase in visitors, which was considered to be the highest increase of any region in the world. This comes even after the region attracted a record setting 26.3 million tourists in 2014 (CTO 2015). The tourism industry is known to be vulnerable to health, safety, security, and environmental challenges, and therefore, FBD may impact negatively on the image and sustainability of this industry with potential visitors becoming reluctant to travel to regions where there is a known or perceived risk of becoming infected with FBD (Paredes et al. 2000; Ehrenberg and Ault 2005; Indar 2015). In the Caribbean, FBD outbreaks are frequently registered by the health authorities and many are known to involve tourists and to be associated with high economic losses to the tourism operators (Indar 2015). Additionally, there are some studies concerning the epidemiology of travel-related pathogens indicating that traveling to the Caribbean region (and also to other regions of the world) may represent a high risk for acquiring foodborne pathogens, *Campylobacter*, *Salmonella*, and *Shigella* in US travelers to the Caribbean (Kendall et al. 2012); *Salmonella* enteritidis in Ontario, Canada, travelers (Tighe et al. 2012); and *Campylobacter* in Dutch travelers (Mughini-Grass et al. 2014). More recently, the prevalence of Shiga toxin-producing *Shigella* species isolated from French travelers returning from the Caribbean was assessed (Gray et al. 2015), providing evidence of the emergence of Shiga toxin-producing *Shigella* on the island of Hispaniola (Haiti and Dominican Republic). This study demonstrates that travelers are capable of spreading these particular *Shigella* strains globally, expanding the risk to a broader level. This is a finding that can be extrapolated to other travel-related pathogens.

### Bacteria prevalence studies in food-producing animals and food products of animal origin in the Caribbean

#### *Salmonella* spp.

The prevalence of *Salmonella* spp. in food-producing animals and food products of animal origin in the Caribbean was reported in 14 studies published between 1995 and 2015. Twelve of these studies were conducted in Trinidad, one in Barbados and one in Trinidad, Grenada, and St. Lucia simultaneously. As seen in Table 3, two studies concerned livestock and poultry, one concerned milk and one concerned raw poultry; five studies concerned layer farms and eggs; and finally, six studies concerned ready-to-eat meats. Prevalence of this bacteria in fecal samples of live animals (livestock and ducks) was low, with values of 2.3–2.7 % in livestock (Adesiyun et al. 2001) and 4.55 % in ducks (Rampersad et al. 2008). In layer farms and eggs, two studies indicated prevalences ranging from 3.8 to 4.66 % on egg shells and 1.2–7.6 % in egg contents (Indar et al. 1998; Adesiyun et al. 2005); 40–73 % of layer farms were positive for *Salmonella* which was revealed in three other studies (Aimey et al. 2013; Adesiyun et al. 2014a, b). The only study conducted on milk detected *Salmonella* in 1.7 % of the samples (Adesiyun et al. 1996), while in raw poultry, *Salmonella* was present in 7.3 % of the carcasses (Rodrigo et al. 2006). In ready-to-eat meat products, *Salmonella* was present in black pudding varying from 3.4 to 14.4 % in the three studies conducted on this food type (Adesiyun and Balbirsingh 1996; Adesiyun 1995; Adesiyun and Benjamin 1996). In ready-to-eat meat-processing plants, *Salmonella* was detected only in pre-cooked meat in one of the studies (Syne et al. 2013), and in a more recent study focused on ready-to-eat meat available in supermarkets, *Salmonella* was not detected in a total of 480 samples (Syne et al. 2015). Although serovar or molecular data are limited, apparently, there is a predominance of *Salmonella* enteritidis in samples from layer farms and eggs, while in ready-to-eat meat products, *Salmonella* agona and *Salmonella* derby are the predominant serovars (Table 3). Although *Salmonella* enteritidis showed predominance in the human isolates in Trinidad and Tobago (Indar et al. 2015a) and in eggs sampled for the studies conducted in the same country (Adesiyun et al. 2005), there are no studies providing information on the issue of serovars. Data from the PulseNet Latin America and Caribbean Network (Campos et al. 2012) showed a high genetic diversity among the strains of *Salmonella* typhimurium, *Salmonella* enteritidis, and *Salmonella* typhi (ST) human isolates circulating in six countries of the region.

**Table 3:** Investigations on *Salmonella* prevalence in food-producing animals and animal source food products in the Caribbean 1995–2015, presented by livestock/food type

Study date	Study location	Sampling site	Species, sample type	Sample size	*Salmonella* prevalence; observations	*Salmonella* serovars	Other bacteria	Reference
Livestock								
2001	Trinidad	Cattle farms, pig farms, sheep farms, goat farms	Fecal samples and rectal swabs from diarrhoeic and non-diarrhoeic animals: bovine, porcine, ovine, caprine	188 diarrhoeic animals, 174 non-diarrhoeic animals	Diarrhoeic animals 5 (2.7 %), non-diarrhoeic animals 4 (2.3 %)	Not indicated	Diarrhoeic animals 140 (74.5 %) *E. coli* 54 (28.7 %) *Campylobacter* spp. Negative for *Yersinia enterocolitica* Non-diarrhoeic animals 119 (68.4 %) *E. coli* 42 (24.1 %) *Campylobacte*r spp. Negative for *Yersinia*	Adesiyun et al. 2001
Poultry—ducks								
2008	Trinidad and Tobago	Muscovy ducks	Form March–September 2003 Fecal samples at small scale and “backyard” farms	110 pooled fecal samples collected from 86 farms	5/110 (4.55 %) positive for *Salmonella*, by bacterial culture 44/110 (40 %) positive for *Salmonella*, by PCR	Kiambu, Orion, Uganda Group E1 (no further characterized)	No other bacteria tested	Rampersad et al. 2008
Layer farms and eggs								
1998	Trinidad	Layer farms and eggs	Eggs—contents and surface samples	750 fresh eggs, 10 farms	Eggs contents 1.2 %, eggs shells 4.66 %	Isolates from the contents Enteritidis (0.8 %) Typhimurium (0.4 %) Isolates from the shells Typhimurium (3.06 %) Enteritidis (0.67 %) Ohio (0.27 %) Cerro (0.27) Infantis (0.27 %) Heidelberg (0.13 %)	No other bacteria tested	Indar et al. 1998
2005	Trinidad	Layer farms and eggs	Composite samples of -pooled eggs from 23 poultry layer farms -6 pooled eggs from 14 shopping malls -6 pooled eggs from 102 other retailers across Trinidad	184 samples: swabs of eggs shells and egg content	Total 24 (13 %) Egg shell samples 7 (3.8 %) Egg content 14 (7.6 %)	Enteritidis in 56.8 % of the *Salmonella* isolates (14/24) Other serotypes Mbandaka, Javiana, Caracas, Ohio, Braenderup, Georgia Group C1	68 (37 %) *E. coli* (52–28.3 % egg shell, 7–3.8 % egg content) 2 (1.1 %) *Campylobacter* (both from egg contents) Negative for *Listeria* spp.	Adesiyun et al. 2005
2013	Barbados	Layer farms and eggs	Eggs; environmental samples from floor pens and from cages (feces, dust, feed, bedding, surface swabs)	26 farms; overall pens 52 and eggs 1059; environmental 491	73 % farms *Salmonella* positive (19/26) Eggs 120/1059 (1.13 %) Environmental 69/491 (14 %)	11 serotypes, predominant enteritidis; other Rubislaw, Isangi, Mbandaka, idikan, Ohio, Alachua, Kentucky, Weltevreden, Typhimurium, Seftenberg	No other bacteria tested	Aimey et al. 2013
2014	Trinidad and Tobago, Grenada, St. Lucia	Layer farms, hatcheries, ports of entry, kitchens of hotels	Cloacal swabs; environmental swabs of deep litters, freshly laid eggs, and poultry feeds	35 farms total, cloacal swabs of layers, environmental swabs, and feed (total) 530 Trinidad and Tobago, 271 Grenada, 488 St. Lucia, pooled samples of freshly laid eggs, 24 (144 eggs) Trinidad, 12 (72 eggs) from Grenada, and 23 (138 eggs) from St. Lucia	14 (40 %) farms positive for *Salmonella* Farms in Trinidad and Tobago 15/530 (2.8 %) Farms in Grenada 4/271 (1.5 %) and Farms in St. Lucia 12/488 (2.5 %) Pooled eggs in Trinidad 3/24 (12.5 %) All the other pooled eggs were *Salmonella* negative	35 isolates, 15 serotypes Anatum, Mbandaka, Polyvalent A neg, Montevideo Groups I-O (I) Enteritidis, Group E, Caracas, Braenderup, Uganda, Muenster, Group C, Group D, Kentucky, Group B Enteritidis represented 6.6 % of the 15 serotypes	No other bacteria tested	Adesiyun et al., 2014 a,b
Milk								
1996	Trinidad	Dairy farms	Bulk milk cow’s feces, and effluents of dairy farms	177 bulk milk, 333 fecal, 168 effluent	1.7 % bulk milk (3 samples) 0.9 % feces (3 samples) 0.6 % effluent (1 sample)	All isolates serotype S. Agona	Bulk milk samples *L. monocytogenes* 3 (1.7 %) *Y. enterocolitica* 2 (1.1 %) *Campylobacter* negative Fecal samples *L. monocytogenes*, 11 (3.3 %) *Campylobacter* spp. 19 (5.7 %) Effluent samples *Campylobacter* spp. 7 (4.2 %) Negative for *Yersinia* or *Listeria*	Adesiyun et al. 1996
Raw poultry								
2006	Trinidad	Poultry and poultry giblets form Retail processors	Broiler carcasses, drip and chicken offal (liver, heart, gizzard) collected from retail processor located in six different health districts across Trinidad	96 carcass samples, 96 drip samples, liver and heart samples from 95 carcasses, 93 gizzard samples	Carcass 7.3 % Drip 3.1 % Gizzard 2.1 % Liver 1 %	Three serotypes Kiambu (53.8 %), Kentucky (38.5 %), Mbandaka (7.7 %)	Carcass and drip samples 100 % positive for aerobic bacteria, *E. coli*, total coliforms, and *Staphylococci* *Campylobacter* spp. (86/96) 89.6 % Liver and heart samples 100 % positive for aerobic and *Staphylococci* *E. coli* ranged from 75 to 100 % depending on health divisions *Campylobacter* 89.6 % Gizzard samples 100 % positive for aerobic bacteria, *E. coli*, total coliforms and *Staphylococci* *Campylobacter* 89.2 %	Rodrigo et al. 2006
Ready-to-eat meat products								
1994	Trinidad	Local vendors and supermarkets	15 outlets selling black puddings and 22 vendors	100 black pudding samples each from supermarkets and street vendors	Supermarkets 5/100 (5 %) Local vendors 11 (11 %)	S. Agona (8) S. Derby (6) S. Ohio (6) S. unnamed (4,12:d-) (11)	Supermarkets *Staphylococcus aureus* 40/40 (100 %) (9, 22.5 % enterotoxinogenic strains) *E. coli* 56/100 (56 %) (1/45 O157:H7 strain) Local vendors *Staphylococcus aureus* 40/40 (100 %) (18, 45 % enterotoxinogenic strains) *E. coli* 79/100 (79 %) (9/66 O157:H7 strain)	Adesiyun and Balbirsingh 1996
1995	Trinidad	10 types of food, predominantly of animal origin and drinks purchased from sale outlets ore hawkers across Trinidad	293 samples of the 10 different types of local foods and drinks	Ice cream (66), black pudding (37), doubles (32), local sweets (29), snow cones (26), preserved fruits (25), roti (25), chutney (20), punch drinks (18), seamoss (15)	*Salmonella* spp.—10 (3.4 %)—only present in one type of food (black pudding)	S. Agona (4) S. Derby (3) S. Albany (2) S. Mbandaka (1)	*Staphylococcus aureus*—58 (27.3 %); highest prevalence in one type of food (black pudding) (27/58) *E. coli*—30 (10.2 %) highest prevalence in one type of food (black pudding) (16/30) Termophilic *Campylobacter* spp.—all samples were negative All isolates of *E. coli* were negative for LT and VT 37.9 % of the *S. aureus* isolates were enterotoxinogenic	Adesiyun 1995
1996	Trinidad	Local abattoir (San Juan) and product processors	Samples of major black pudding ingredients and ready-to-eat black pudding collected from all processors	125 pre-heated and post-processed samples	18/125 (14.4 %)	S. Agona (94 % of the isolates) S. Derby	*Staphylococcus aureus* 121/125 (96.8 %) *E. coli* 107/125 (85.6 %) *Campylobacter* spp. 5/125 (4 %)	Adesiyun and Benjamin 1996
2013	Trinidad	Ready-to-eat meat-processing plant	Chicken franks, chicken bologna, bacon, factory water, surfaces, and air	24 each of raw meat, uncooked formulated products, cooked products, air 48 raw non-meat ingredients 50 swabs of surfaces	Overall prevalence was 2.9 % (5 of 170); only detected in pre-cooked meat during the production of chicken franks and chicken bologna 11 and 16.7 %, respectively	One *Typhimurium* from raw meat used for producing chicken franks One-group C3 from flaked meat; two isolates recovered from pre-processed meat during production of chicken bologna, group C1 and C3 One isolate group B found after flaking	*Staphylococcus aureus* (most detected pathogen in precooked samples; also in unacceptable levels in post cooked samples); 50 % (10/20) precooked mixtures of bacon and bologna contaminated with *Listeria* spp., including 4 *L. monocytogenes*; *E. coli* present in pre-cooked mixtures of franks and bologna Environmental samples positive for *S. aureus*	Syne et al. 2013
2015	Trinidad	Ready-to-eat meats from eight supermarkets across Trinidad	Samples of locally processed meats (chicken franks, chicken bologna, bacon)	480	All 480 samples were negative for *Salmonella*		1.5 % *E. coli* 10 % *S. aureus* *Listeria* spp. 19.4 % *L. monocytogenes* 7.5 %	Syne et al. 2015

#### *Campylobacter* spp.

Publications on the prevalence of *Campylobacter* spp. refer to studies conducted in Trinidad, Barbados, and Grenada. Only four studies were aimed at specifically isolating *Campylobacter* in livestock and food products of animal origin (Table 4), while there are other studies that reveal the presence of *Campylobacter* together with other pathogenic bacteria as seen in Table 3 and also in Table 4. Overall, the prevalence of *Campylobacter* is high in poultry, with values of about 90 % for the analyzed samples, carcass 89.6 %, liver and heart 89.6 %, gizzard 89.2 % (Rodrigo et al. 2006), cloacal swabs 94.2 % (Workman et al. 2005), and ceca 72 % (Miller et al. 2009)–93.5 % (Hariharan et al. 2009). For chicken layers, two studies indicated prevalences of 22 % (Miller et al. 2009) and 56.3 % (Hariharan et al. 2009). Prevalence in pigs varied from 20 % in carcass swabs to 54 % in rectal swabs (Adesiyun and Krishnan 1995) and 11.8 % (Gibbons et al. 2006) to 90.5 % (Workman et al. 2005) in feces. A study conducted in feces and rectal swabs from cattle, pigs, sheep, and goats indicated the overall prevalence of *Campylobacter* to be 28.7 % in diarrheic animals and 24.1 % in non-diarrheic animals (Adesiyun et al. 2001). Low-prevalence figures were detected in sheep feces (4.2 %) (Workman et al. 2005), goat rectal swabs (3.7 %) (Stone et al. 2014), and dairy cattle feces (ranging from 0 to 5.7 %) (Workman et al. 2005; Adesiyun et al. 1996, respectively). Regarding meat, Workman et al. (2005) recovered *Campylobacter* from the following: chicken 58.4 %, pork 3.9 %, beef 1.8 %, and turkey 5 %. In ready-to-eat meat products, *Campylobacter* was found in black pudding (4 %; Adesiyun and Benjamin 1996) and together with other pathogens in processed ready-to-eat meat products in a meat-processing plant associated with a recall of the following three processed meat products: chicken franks, ready-to-eat spice ham, and turkey ham (Gibbons et al. 2006). Studies also indicate a general higher frequency of *Campylobacter coli* than *Campylobacter jejuni*. Detailed information is given in Tables 3 and 4. Regarding the epidemiology of *C. jejuni* and C. *coli*, there is a reference on the typing of these two isolates, but this study was mainly intended to assess the benefits and limitations of different typing approaches, rather than an epidemiological study (Behringer et al. 2011). Previous to this, Workman et al. (2005) investigated the sources of this bacteria in a poultry production and packing operation in Barbados finding 10 distinct RAPD genotypes and no evidence of vertical transmission of *Campylobacter* in the flock, suggesting that it might have been contaminated from more than one source in the farm environment under investigation. Also, Miller et al. (2009) performed DNA identification of *C. jejuni* and *C. coli* from poultry in Grenada using combined typing methods, finding phylogenetic associations with previously described poultry-related strains.

**Table 4:** Investigations on *Campylobacter* spp. prevalence in food-producing animals and animal source food products in the Caribbean 1995–2015, presented chronologically

Study date	Study location	Sampling site	Species, sample type and size	*Campylobacter* spp. prevalence and occurrence of other pathogenic bacteria	Reference
1995	Trinidad	A local abattoir (San Juan)—pig slaughter	141 samples each of pig rectal and tongue swabs and 150 tonsillar samples for *Yersinia enterocolitica* detection. Rectal swabs also cultured for *Listeria* and *Campylobacter* detection; 155 carcass swabs were included	*Campylobacter* Rectal swabs 54 % (75/139), 72/139 (51.8 %) positive for *C. coli*, 3 (2.2 %) positive for *C. jejuni* Carcass swabs 20 % (31/155), 27/155 (17.4 %) positive for *C. coli* 4/155 (2.6 %) positive for *C. jejuni* *Yersinia enterocolitica* 23/141 (16.1 %) and 9 (6.4 %) rectal and tongue swabs, from tonsillar samples; 7.3 % (11/150) were positive; all isolates were serotype O:3 *Listeria monocytogenes* 7/139 (5.0 %) of rectal swabs; 3/155 (1.9 %) of carcass swabs All *L. monocytogenes* isolates were serotype 4.	Adesiyun and Krishnan 1995
2003	Trinidad	Meat-processing plantassociated with a previous recall of three processed meat products (chicken franks, spice ham, and turkey ham ready-to eat) as a result of contamination by *L. monocytogenes* in 2003	Raw (11) and processed ready-to-eat meat products (32), air samples (4) food contact surfaces (10), fecal (34), and effluent (17) samples from the piggery located in close proximity to the plant; second sampling after implementation of sanitizing program on 5 raw products and 5 finished products	*Campylobacter* spp. 10/17 (58.8 %) effluent samples and 4/34 (11.8 %) fecal samples *Salmonella* negative for all air and food contact samples 11/32 (34.4 %) processed ready-to-eat samples positive for *Salmonella* and *E. coli*, *Listeria* spp., and *Campylobacter* spp., in combination or singly (not specified) *Listeria* spp., *E.* coli, *Campylobacter* negative for all air and food contact samples *E. coli* and *Listeria* spp 10/11 (90.9 %) raw meat products 11/32 (34.4 %) processed ready-to-eat samples positive for *Salmonella* and *E. coli*, *Listeria* spp., and *Campylobacter* spp., in combination or singly (not specified) After the sanitizing implementation *Listeria* 4/5 (80 %) raw products and 1/5 (20.0 %) finished products	Gibbons et al. 2006
2005	Barbados	Food animals were tested either while being reared on the farm or upon delivery to the abattoir for slaughter. On farms, fresh feces were collected from pigs, cows, and sheep Swabs from dogs and cats from the local Royal Society for the Prevention of Cruelty to Animals (RSPCA), a private veterinary clinic, and various households. Samples from wild birds collected at a recreational shooting swamp. The wild birds tested included both migratory and resident birds; mongooses were trapped for an independent study being carried out by the Centere for Resource Management and Environmental Studies of the University of the West Indies. Monkeys (*Cercopithecus aethiops sabaeus*) were processed by the Barbados Primate Research Centre and Wildlife Reserve	Fecal specimens of 596 animals and 311 samples of animal food products Rectal swabs were obtained from 130 healthy and diarrheic dogs and 51 cats; cloacal swabs were obtained from 69 broiler chicks and from 61 wild birds Rectal swabs were also obtained from 9 mongooses and from 70 vervet monkeys (*Cercopithecus aethiops sabaeus*) Various cuts of a range of fresh meats were purchased from 10 retail outlets on the island, ranging from small meat shops to large supermarkets Fresh, raw goat’s milk and fresh, pasteurized cow’s milk were also analyzed for *Campylobacter* contamination Tap water was tested on a monthly basis	462 (51 %) isolates of *Campylobacter* were recovered from animals and animal food products Chickens 94.2 %, pigs 90.5 %, dogs 46.9 %, cats 37.3 %, wild birds 39.3 %, monkeys 17.1 %, sheep 4.2 %, cows 0 % Chicken meat 58.4 %, pork 3.9 %, beef 1.8 %, turkey 5 %. *C. jejuni* was the most commonly identified species isolated from dogs, chickens, and chicken meat Pigs carried *C. coli* almost exclusively, *C. upsaliensis* was only isolated from dogs and cats, and *C. helveticus* was recovered from cats alone. UPTC strains were harbored exclusively by several wild birds	Workman et al. 2005
2006–2007	Grenada	Five chicken farms, 6-week broilers, and 72 weeks of age layers	Samples collected at each farm during processing (manually) ceca and layers; total 60 samples	93.3 % (56/60—43 isolated from broilers, 13 isolated from layers) *Campylobacter* jejuni 7 (12.5 %) *Campylobacter* coli 49 (87.5 %)	Miller et al. 2009
2006–2007	Grenada	10 chicken farms	Ceca samples from 125 chickens—77 broilers and 48 layers	79 % of the chickens positive; isolation rate of 93.5 % for broilers and 56.3 % for layers	Hariharan et al. 2009
2011	Grenada	21 family production units of sheep and goats from 6 parishes of Grenada and 8 production units from the island of Carriacou	Rectal swabs from 155 sheep and 252 goats. All animals clinically health and >6 month old	*Campylobacter* spp. 15 (3.7 %), of which *C. jejuni* 3.2 % in sheep and 3.1 % in goats *C. coli* 1.3 % in sheep and 0 % in goats Genotype study suggested sheep and goat clones specific to Grenada	Stone et al. 2014

#### Verotoxigenic *E. coli* and other pathogenic zoonotic bacteria

The presence of *E. coli* and also of other pathogenic zoonotic bacteria in livestock and food products of animal origin in the Caribbean region is shown in some of the previous mentioned studies, where apart from *Salmonella* spp. and/or *Campylobacter* spp., the authors broadened their research to other pathogenic bacteria (Tables 3 and 4).

Addressing the presence of *E. coli*, there was one study aimed at specifically determining the presence of verocytotoxigenic *E. coli* (VTEC) in dairy farms in Trinidad—*E. coli* was isolated from all analyzed sources (fecal samples collected from milking cows, calves, and humans; rectal swabs obtained from pet farm dogs; and bulk milk as well as effluent samples from the milking parlor) with 61 isolates found positive for VTEC, 16.6 % from cows, 14.6 % from calves, 3.2 % from farm dogs, and 7.1 % from humans (Roopnarine et al. 2007). Another study also conducted in Trinidad (Table 3) indicated the presence of this microorganism in livestock with a 74.5 and 68.4 % occurrence in diarrhoeic and non-diarrhoeic animals, respectively (Adesiyun et al. 2001). In the same country, verotoxigenic *E. coli* was detected in 37 % of tested eggs (Adesiyun et al. 2005) and in 75–100 % of raw poultry samples. Investigations of ready-to-eat meat products in Trinidad indicated *E. coli* levels of 1.5 % (Syne et al. 2015), 10.2 % (Adesiyun 1995), 34 % (Gibbons et al. 2006), 56–79 % (Adesiyun and Balbirsingh 1996), and 85.6 % (Adesiyun and Benjamin 1996). Very few of these studies tested *E. coli* for VTEC (Table 3).

Other bacteria that are mentioned in the searched literature include *Listeria* spp., *Yersinia* spp., and *S. aureus*. In pig samples at slaughter, *Listeria monocytogenes* was found in 5 % of the rectal swabs and 1.9 % of the carcass swabs and *Yersinia enterocolitica* in 16.1 and 6.4 % of the rectal and tongue swabs, respectively, and from 7.3 % of the tonsillar samples (Adesiyun and Krishnan 1995). These two bacteria were also detected in bulk milk samples, 1.7 and 1.1 %, respectively, and *L. monocytogenes* was found in fecal samples of cows in the same study (Adesiyun et al. 1996). *Listeria* spp. is referred to in studies conducted in ready-to-eat meat products, with levels of 18.3 % (Syne et al. 2013) and 19.4 % (Syne et al. 2015). Finally, regarding *S. aureus* prevalence, it has been found in milk or milk-related environments in Trinidad, 100 % of the 287 milk samples (Adesiyun et al. 1995), raw poultry with all carcass (96), drip (96), liver and heart (95), and gizzard (93) samples positive for this bacteria (Rodrigo et al. 2006). Regarding ready-to-eat meat products, *S. aureus* was present in 100 % of the 80 black pudding samples acquired in 1994 in both supermarkets and street vendors in Trinidad (Adesiyun and Balbirsingh 1996) and in 27.3 % of the 293 ready-to-eat samples, including black pudding collected 1 year later in the same country (Adesiyun 1995), with a higher presence in the black pudding samples (27/58) and again later in 96.8 % of the 125 black pudding samples also (Adesiyun and Benjamin 1996). Two additional studies conducted in Trinidad in 2013 and 2015 reveal the presence of *S. aureus*, 27.1 % (46/170) in samples of air, food, and environment of a large meat-processing plant, being present in both pre-cooked and post-cooked meat samples, and in 10 % of the 480 ready-to-eat samples collected at different supermarkets (Syne et al. 2013, 2015) (Table 3). Enterotoxinogenic strains were determined in only two of the above mentioned studies, with 33.8 and 37.9 % positive out of the isolated strains (Adesiyun 1995; Adesiyun and Balbirsingh 1996).

Overall, bacteria prevalence studies in food-producing animals and food products of animal origin mentioned in this review have limited significance. During the past 20 years, only a few sporadic studies were published, mostly related to investigations on layer farms and eggs and ready-to-eat meat products conducted in only three of the Caribbean countries (Trinidad, Barbados, Grenada), which makes it difficult to determine regional trends. Another limitation relates to the diverse sampling plans and methodological steps followed in the detection and identification of the microorganisms in these studies, which make it difficult to compare and interpret the findings.

### Sources of animal and food contamination

Many foods of both animal and plant origin can be contaminated by disease-producing microorganisms at one or more stages of food production, beginning on the farm and ending in the home. While livestock and other domestic animal sources are often implicated, the ultimate sources of contaminants can extend back to infected feral or wild animals (Adesiyun et al. 1998; Hulebak et al. 2013). Several wildlife species living in the Caribbean are recognized as carriers and shedders of the pathogens addressed in this overview. Roach et al. (2003) found *Salmonella* and *Shigella* in captive African green monkeys (*Chlorocebus aethiops*) in Barbados with 16 and 32 of 1608 monkeys found positive for *Salmonella* spp. and *Shigella sonnei*, respectively. In St. Kitts and Nevis, an outbreak of *Yersinia enterocolitica* was registered in captive green monkeys (Soto et al. 2013). Mongoose—small Asian mongoose (*Herpestes javanicus*) and small Indian mongoose (*Herpestes auropunctatus*)—are recognized as carriers and shedders of *Salmonella* spp. as seen in Grenada (Miller et al. 2014, 2015) and thermophilic *Campylobacter* spp. as observed in Barbados (Rhynd et al. 2014). Wild rats (*Rattus* spp.) have also been shown to be carriers of *Salmonella* spp., *Campylobacter* spp., and *E. coli* (Nkogwe et al. 2011). Other animals species found in the Caribbean and seen as carriers of FBD that include the blue land crab (*Cardisoma guanhumi*), commonly used as a food item in the Caribbean region that has been shown to harbor *E. coli* and *Salmonella* (Amadi et al. 2015) and wild and domestic green iguanas (*Iguana iguana*) used as food for example in Grenada, may serve as a reservoir of *Salmonella* (Sylvester et al. 2013). Finally, cane toads (*Bufo marinus*) were found with high levels of *Salmonella* and harboring serovars that are emerging as causes of human disease in the western hemisphere (Drake et al. 2013). In spite of this compilation of possible wild animal contamination sources, no study was found attempting to correlate rates of infection in wild animals and livestock animals in the region. Only a small number of studies were found correlating rates of infection in live animals and food contamination (Adesiyun et al. 2014a, b), and only a few others provide specific data on food attribution with FBD in the Caribbean as previously mentioned.

### The need for integrated animal-human surveillance of zoonotic foodborne pathogens

It is recognized that in order to assure the safety of food of animal origin, zoonotic pathogens in foods have to be controlled through a continuous farm to fork system, which should take into account not only the assessment of risks but also technical possibilities, consumers’ attitude/behaviors, and cost-benefit analysis (Nastasijević 2000). Several studies recognize the need for effective surveillance systems to identify sources of disease (Nastasijević 2000; Pires et al. 2011) and the integration of animal health, food pathogen, and foodborne disease surveillance globally and especially in developing and in-transition countries (de Balogh et al. 2013; Vidal et al. 2013) and specifically in the Americas and the Caribbean region (PercedoAbreu et al. 2011; Hulebak et al. 2013; Gebreyes et al. 2014). Effective surveillance of animal diseases is in fact a priority for the World Organization of Animal Health (OIE) and Food and Agriculture Organization of the United Nations (FAO) (Corning 2014), and tools are available nowadays to prioritize zoonoses using a One Health perspective in a way to minimize fiscal and personal limitations as well as the absence of comprehensive prevalence data. These can be used by human and animal health agencies to coordinate across sectors in a more effective response to zoonotic diseases (Rist et al. 2014).

In the Caribbean, interactions between medical, wildlife, and veterinary professionals are currently considered very limited. The low internal capacity of Caribbean governments, their limited resources, and the high burden of infectious human and animal disease across the Caribbean region are recognized and point toward the relevance of pursuing a holistic One Health approach, with interdisciplinary engagement on a regional scale (ACP 2013). To overcome these limitations, national and regional organizations such as the Caribbean Animal Health Network (CaribVET) are increasingly committed to implementing or improving established surveillance systems according to a One Health approach. Results from a survey conducted by CaribVET in 2009 “Prevention of contamination of the human food chain” was identified as the most important area for the application of risk assessment methodologies to select animal health issues (Percedo Abreu 2011). This result was correlated with the influence of environmental characteristics of the Caribbean tropical area and the growing importance of tourism in the islands. Regarding this last aspect, response surveillance systems are also identified and under preparation for hotels and cruise ships to address health, safety, and environmental issues that pose serious threats to the sustainability of tourism in the region (Indar 2015). There are also environmental health initiatives with the Caribbean EcoHealth Program (CEHP) being carried out to enhance integrated research and capacity development (Forde et al. 2011).

Contributing to an overall human-animal health surveillance strategy, starting at a regional level and in an animal health research perspective, several approaches could be undertaken by each country to gather relevant information to be used in targeting actions in order to improve food safety. Addressing food of animal origin under a farm to the fork approach research strategy, we suggest data collection on the following points (an initial prioritization of zoonoses could be considered):

- Systematic examination of potential hazards that exist in each stage of the meat and other food animal products (milk, eggs) chain and to determine their epidemiological significance;
- Evaluate and manage hygiene and sanitary control systems on farms and at food processing sites;
- Determine sources of animal and food contamination, including wildlife;
- Explore food safety practices and perceptions of consumers.

The outcomes of these research studies could then be used to develop interventions aimed at protecting food animals from infections and reduce dissemination of foodborne hazards.

## Conclusions

Livestock is very important economic and cultural sectors in the Caribbean. Future projections indicate changes in the dietary habits of local populations, a considerable increase in meat consumption and rising demand for other animal products such as eggs. Presently, studies limited to a few countries in the Caribbean indicate an apparent high prevalence of *Campylobacter* and *E. coli* in eggs and chicken and a low prevalence of *Salmonella* spp. in food-producing animals and food products of animal origin. Further research is needed to identify which animals and foodstuffs are the main sources of infections. FBDs are a substantial concern in the region, and *Salmonella* spp., *Shigella* spp., and *Campylobacter* spp. are the main causative bacteria of these incidences, with *Salmonella* showing a rise in reported human cases over the past 20 years. Therefore, further epidemiological studies are needed to establish possible correlations between human and animal diseases that could lead to the establishment of preventive measures. These will ultimately benefit not only the health of populations but would also contribute to increasing tourists’ confidence in visiting the Caribbean region. From the animal health regional perspective, further research measures could contribute with relevant data to establish or improve existent integrated surveillance systems to assure the safety of food of animal origin.
